# Diversity of *Haemaphysalis*-associated piroplasms of ruminants in Central-Eastern Europe, Hungary

**DOI:** 10.1186/s13071-015-1236-0

**Published:** 2015-12-09

**Authors:** Sándor Hornok, Nóra Takács, Jenő Kontschán, Zsolt György, Attila Micsutka, Serena Iceton, Barbara Flaisz, Róbert Farkas, Regina Hofmann-Lehmann

**Affiliations:** Department of Parasitology and Zoology, Faculty of Veterinary Science, Szent István University, Budapest, Hungary; Plant Protection Institute, Centre for Agricultural Research, Hungarian Academy of Sciences, Budapest, Hungary; Veterinary Authority, Pásztó, Hungary; Clinical Laboratory, Vetsuisse Faculty, University of Zurich, Zurich, Switzerland

**Keywords:** *Babesia*, *Theileria*, Tick, Cattle, Small ruminants, Cervids

## Abstract

**Background:**

Increasing numbers of genetic variants are being recognized among piroplasms, but the precise taxonomical status, the tick vector and the geographical range of several species or genotypes are still unknown. Bovine piroplasmosis was reported to re-emerge in north-east Hungary. Because *Theileria*-infection was newly diagnosed in one cattle herd in the same region of the country, the aim of this study was to molecularly identify the relevant agent, to find its local vector tick species, and to examine the range of *Babesia*/*Theileria* spp. of ruminants in *Haemaphysalis* sp. ticks collected previously in Hungary.

**Findings:**

Blood samples were drawn on two occasions from 90 dairy cattle in northern Hungary, and ticks were collected on their pastures. In addition, questing ticks (315 *Haemaphysalis inermis*, 259 *H. concinna* and 22 *H. punctata*), which originated mainly in the same region of the country from 2007, were included in the study. DNA was extracted from these samples, followed by molecular analysis for piroplasms.

In the cattle *Theileria orientalis* was identified, with 100 % sequence homology to isolates from Japan, China, South-Africa and Australia. Based on GenBank data this genotype has not been previously reported in Europe. The prevalence of infection in the herd remained almost constant in the main tick season, suggesting exposure in previous years.

Retrospective analysis of ticks revealed the presence of *Babesia crassa* in *H. inermis*, for the first time in Europe and in this tick species. On the other hand, *H. concinna* carried five different piroplasms, including *B. motasi* that was also newly detected in Central-Eastern Europe and in this tick species; whereas *H. punctata* harboured *Theileria* sp. OT3, hitherto known to occur in the Mediterranean region.

**Conclusions:**

Results of this study broaden the range of piroplasms that are infective for ruminants in Central-Eastern Europe. Although bovine babesiosis and theileriosis was known to occur in Hungary, molecular evidence is provided here for the first time on the presence of *Babesia* and/or *Theileria* spp. of sheep, goats and cervids in Hungary.

## Findings

### Background

Piroplasms (Apicomplexa: Piroplasmida) are unicellular, tick-borne parasites that infect blood cells of vertebrates. In this category both the genus *Babesia* and the genus *Theileria* appear to be geographically widespread [1], affecting domesticated and game animal species, even humans, according to their lower or higher pathogenicity.

Piroplasms represent an unfolding field of research in parasitology, because *Babesia* and *Theileria* spp. are not as host-specific as previously thought [2] and the taxonomy of several species has been revised (e.g. [3]). Apart from this, a steadily increasing number of new species (e.g. *Babesia vulpes*: [4]) and genotypes [5] are being molecularly characterized, the latter frequently with unknown pathogenicity.

In this context, it is also evident that babesioses and/or theilerioses may show a changing epidemiological situation, including regionally disappearing and emerging species. In particular, it was reported that *B. divergens* is becoming extinct in north-east Hungary [6], and *B. major* and *T. buffeli* were newly identified in the same region [7]. Recently, during routine haematological examination, further cattle herds were recognized to harbour piroplasms in the same region of the country. Therefore the present study was initiated in order to identify any relevant agents and to uncover the range of *Babesia* and *Theileria* species in the local tick population. Because *B. major*, members of the *T. orientalis* complex and several other piroplasms of ruminants are transmitted by *Haemaphysalis* spp. in a worldwide context [8–10], the molecular investigation of potentially present *Babesia* and *Theileria* species focused on this hard tick genus.

## Methods

Individual, EDTA-anticoagulated blood samples were collected by coccygeal venipuncture in a dairy cattle herd in which piroplasms had been detected microscopically during routine haematological examination of asymptomatic cattle. The herd consisted of 90 cows and their calves that are kept extensively (grazing pastures from April until November) in northern Hungary (Nógrád county, Pásztó, geographical coordinates: 47° 55′ 26.9″ N, 19° 42′ 21.4″ E). All cows were blood-sampled prior to the grazing period (April, 2015) and after two months of grazing (June, 2015). In addition, 73 ticks were collected from the pastures with the dragging-flagging method in the main tick season (April, 2015).

The DNA was extracted using QIAamp DNA Mini kit (Qiagen, Hilden, Germany) from all blood samples and ticks as described [7, 11]. The presence of piroplasms was investigated by a conventional PCR that amplifies an approx. 500 bp long part of the 18S rDNA gene of *Babesia*/*Theileria* spp. with the primers BJ1 (forward: 5′-GTC TTG TAA TTG GAA TGA TGG-3′) and BN2 (reverse: 5′-TAG TTT ATG GTT AGG ACT ACG-3′) [7]. This was followed by sequencing 20 PCR positive samples. Phylogenetic analysis was carried out according to the neighbor-joining method as reported [12]. The sequence was submitted to GenBank (KT725847). Exact confidence intervals (CI) for the prevalence rates were calculated at the level of 95 %.

To trace potential vectors of the *T. orientalis* complex and other piroplasms infecting ruminants in the region, a larger number of ticks (596 *Haemaphysalis* specimens, including 315 *H. inermis*, 259 *H. concinna* and 22 *H. punctata*) were also screened. These ticks had been collected from the vegetation by the dragging-flagging method from March to July in 2007, on 20 locations (pastures, forests, meadows) mainly in northern Hungary [13]. DNA extraction from pools of these ticks (Table 1) was done by the MagNA Pure LC total nucleic acid isolation kit (Roche Diagnostics, Rotkreuz, Switzerland), and prior to testing for piroplasms the amplifiable DNA contents of each tick pool were evaluated as reported [14]. These DNA samples were stored at −80 °C until molecular analysis (PCR and sequencing of 24 PCR positive samples) as described above. Sequences were submitted to GenBank (KT725848-54: Table 1).

**Table 1:** Results of molecular analyses of 596 ticks collected in 2007 in Hungary

*Haemaphysalis* species	Tick stage or sex	Number of ticks per pool	PCR positive/all pools	Results of sequencing (length, % identity, sample number)	Location in Hungary^a^	Reference sequence	Accession number of sequence in this study (name of isolate)
*H. inermis*	male	5	7/24	*Babesia crassa* (410 bp, 98.6 %, 4×)	B, P, S	KF791205	KT725848 (Bcr-Hu1)
	female	5	12/40	*Babesia crassa* (410 bp, 98.6 %, 6×)	B, P, S	KF791205	KT725848 (Bcr-Hu1)
*H. concinna*	male	3	10/29	*Babesia* sp. Kh-Hc222 (409 bp, 99.8 %, 1×)	P	KJ486568	KT725849 (BKh-Hu1)
				*Babesia* sp. Irk-Hc133 (409 bp, 100 %, 2×)	B, P, A, C	KJ486563	KT725852 (BIrk-Hu1)
				*Theileria capreoli* (439 bp, 99.8 %, 2×)	B, P	AY726008	KT725850 (Tc-Hu1)
				*Theileria* sp. ZS TO4^b^ (440 bp, 100 %, 1×)	P	DQ520836	KT725851 (TZSTO4-Hu1)
	female	3	5/16	*Babesia motasi* (403 bp, 99.3 %, 1×)	B	AY260179	KT725853 (Bmo-Hu1)
				*Babesia* sp. Irk-Hc133 (409 bp, 100 %, 1×)	B, P, A, C	KJ486563	KT725852 (BIrk-Hu1)
				*Theileria* sp. ZS TO4 (440 bp, 100 %, 1×)	P	DQ520836	KT725851 (TZSTO4-Hu1)
	nymph	10	7/7	*Babesia* sp. Irk-Hc133 (409 bp, 100 %, 1×)	B, P, A, C	KJ486563	KT725852 (BIrk-Hu1)
				*Theileria capreoli* (439 bp, 99.8 %, 1×)	B, P	AY726008	KT725850 (Tc-Hu1)
				*Theileria* sp. ZS TO4 (440 bp, 100 %, 1×)	P	DQ520836	KT725851 (TZSTO4-Hu1)
	larva	52	0/1	-		-	-
*H. punctata*	male	5	1/5	*Theileria* sp. OT3 (160 bp, 100 %, 1×)	P	KF470868	KT725854 (TOT3-Hu1)
	female	3	1/3	*Theileria* sp. OT3 (160 bp, 100 %, 1×)	V	KF470868	KT725854 (TOT3-Hu1)
	nymph	1	0/1	-		-	-

^*a*^ *Abbreviations*: *B* Börzsöny Mountains, *P* Pilis Mountains, *S* Szekszárd Hills, *V* Visegrád Mountains, *A* Ásotthalom, *C* Cserhát Mountains

^b^Note that *Theileria* sp. ZS TO4 and *T. capreoli* are 99.5 % identical, but for comparability with relevant literature data and because of their separate clustering on the phylogentic tree (Fig. 1) both names are maintained in the table and in the text

### Ethical approval

The animals were sampled as part of the regular veterinary care.

## Results and discussion

### Investigation of the current epidemiological situation in a cattle herd

Before grazing 52 of 90 (57.7 %, CI: 46.9–68.1 %), then (after two months) during grazing 51 of 90 (56.6 %, CI: 45.8–67.1 %) cows were PCR positive. Three cows became PCR negative, and two became PCR positive between the two samplings. All the remaining 49 animals had parasitaemias (detectable by PCR) which persisted during the evaluated period. Similarly high rates of infections were reported from other continents (e.g. [15]) and can be explained by long-term persistence of theileriae in the blood stream [16].

**Fig. 1:**
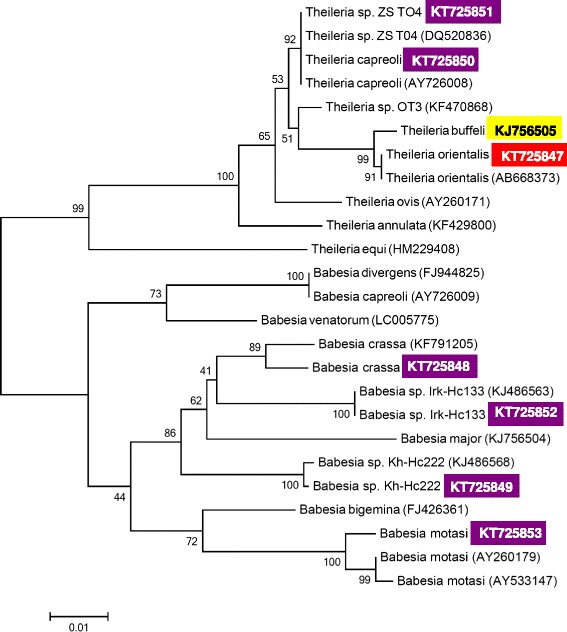
Phylogenetic comparison of partial 18S rDNA sequences of piroplasms identified in Hungary (inverse colour), with related sequences from the GenBank. Inverse purple colour indicates sequences amplified from ticks in the present study; yellow and red colour designate formerly and recently identified *Theileria* isolates from cattle, respectively. The names of piroplasm isolates obtained from ticks in the present study and corresponding lengths of sequences used for the phylogenetic tree are included in Table 1. The sequence of *Theileria* sp. OT3 obtained in this study was considerably shorter than those of other piroplasms, therefore it was excluded from the phylogenetic analysis. Branch lengths correlate to the number of substitutions inferred according to the scale shown. The isolation source and country/continent for reference sequences are the following: AY726008 (roe deer, Europe), KF470868 (sheep, China), DQ520836 (red deer, Poland), AY260171 (sheep, Sudan), KF429800 (cattle, Iran), AB668373 (cattle, Japan), HM229408 (horse, China), KJ486568 (*Haemaphysalis concinna*, Russia), KJ756504 (cattle, Hungary), KF791205 (*H. parva*, Turkey), KJ486563 (*H. concinna*, Russia), FJ944825 (cattle, France), AY726009 (roe deer, France), LC005775 (*Ixodes persulcatus*, Mongolia), FJ426361 (cattle, Brazil), AY260179 (sheep, Netherlands), AY533147 (sheep, Spain)

In all 20 blood samples sequencing revealed the same *Theileria orientalis*/*buffeli* genotype (length: 432 bp), with 100 % identity to *T. orientalis* isolates from Japan (in cattle: AB668373), China (in buffalo: HM538223), South-Africa (in buffalo: GU733374) and Australia (in cattle: AB520953). Based on GenBank data this genotype has not been previously reported in Europe. The sequence was submitted to GenBank (KT725847). This genotype differed with five nucleotides from the *T. buffeli* isolate reported recently from cattle in Hungary (KJ756505: [7]) and these two clustered separately with a high bootstrap support on the phylogenetic tree (Fig. 1).

Ticks collected from the pastures (including all three *Haemaphysalis* spp.) were PCR negative (sample number per species not shown). Thus the local vector of *T. orientalis* could not be identified. Taken together, because in the relevant herd the main tick season in the spring apparently had minor influence on the prevalence of *T. orientalis* infection, it might have originated in previous years.

### Molecular analysis of piroplasms in ticks collected in 2007

Seven piroplasm species or genotypes were shown to be present in these ticks (Table 1), all newly detected in Hungary. *H. concinna* carried five different piroplasms, as contrasted to one found in *H. inermis*, and another in *H. punctata*. The phylogenetic positions of these *Babesia* and *Theileria* spp. and relevant genotypes from GenBank are shown on Fig. 1.

*H. inermis* is the most abundant tick species of its genus in Hungary, and is regarded as an emerging species [13]. In ten pools of *H. inermis* (Table 1) a sequence was identified which had the highest (98.6 %) homology to (6 nucleotide difference from) two Turkish isolates of *B. crassa*: one from sheep (AY260177: [17]) and one from *H. parva* ticks (KF791205: [18]). The *B. crassa* genotype from *H. inermis* in the present study and the one previously reported from *H. parva* clustered together (Fig. 1). *Babesia crassa* has low pathogenicity in small ruminants [19] and its vector is/are most likely *Haemaphysalis* sp./spp., because (apart from *H. parva* as mentioned above) it was isolated from *H. concinna* in China (JX542614) and from *H. sulcata* in Turkey (KF034782) [20]. Until now this species or closely related genotypes were known to occur only in the Middle-East [17].

Another lowly pathogenic species, *B. motasi* was present in *H. concinna*. This piroplasm occurs in Northern, Western and Southern Europe, where it is transmitted by *H. punctata* [21]. To the best of our knowledge, *B. motasi* was not reported previously from *H. concinna*. The first Hungarian *B. motasi* genotype has 99.1–99.3 % sequence similarity to (i.e. three to four nucleotide difference from) isolates found in the Netherlands and in Spain, which differed from each other with two nucleotides, as reflected by the position of these three genotypes on the phylogenetic tree (Fig. 1).

Further piroplasm genotypes demonstrated from *H. concinna* or *H. punctata* in the present study (Table 1) are known to occur in countries close to Hungary (*Theileria* sp. ZS TO4 in red deer, *H. concinna* in Austria: [22]; *T. capreoli* in red deer in Poland: [23]), or in Southern Europe (*Theileria* sp. OT3 in sheep in Italy: [24]). Surprisingly, two *Babesia* genotypes found here in *H. concinna* were formerly reported in Far Eastern Russia (KJ486568 from Khabarovsk) and in East Siberia (KJ486563 from Irkutsk region), in both cases from *H. concinna* ticks [5]. In this way identical (or nearly identical) *Babesia* sequences were found in *H. concinna* ticks from different parts of Eurasia (Hungary, East Siberia, Far East), i.e. the relevant genotypes appear to be geographically very widespread. This may be related to the broad geographical range of *H. concinna* in Eurasia [25], and connectedness of its eastern and western habitats via longitudinal migration of birds [26]. Birds are known to be preferred hosts of this tick species [11, 25].

Taken together, in the present study several new genotypes of *Babesia* and *Theileria* spp. were identified. The high genetic variability of the 18S rDNA gene among piroplasms infecting ruminants is well known [5, 27]. Accordingly, *Babesia* and *Theileria* spp. detected in *Haemaphysalis* sp. ticks of the present study were found to exhibit pronounced heterogeneity in the amplified part of their 18S rDNA gene.

It was also shown here that different *Babesia*/*Theileria* genotypes or species are associated with different *Haemaphysalis* spp. in Central-Eastern Europe (i.e. none of the genovariants were shared between the ticks species analysed here). This is in line with the relatively narrow spectrum of competent vector tick species in the case of piroplasms [28].

Piroplasms with close phylogenetic relationships were reported to have similar host-associations as well [5]. In the present study *H. inermis* and *H. punctata* harboured only one *Babesia* and one *Theileria* sp. of sheep, respectively. In contrast to this, *H. concinna* carried piroplasms of wild cervids, as wells as of domestic small ruminants. The preferred hosts of *H. concinna* are red deer, roe deer, but it can also infest sheep and goats in Central-Eastern Europe [29]. Therefore it is likely that domestic and wild ruminants in the region are exposed to and infected with the detected piroplasms.

The high diversity of piroplasms of ruminants in ticks of the present study support previous literature data, because the number of *Babesia* and *Theileria* genotypes were shown to be highest in *Haemaphysalis* spp. as contrasted to other tick genera [5]. Interestingly, none of the nearly 600 *Haemaphysalis* sp. ticks, collected in 2007 and analysed here, harboured the three piroplasms (two genotypes of *T. orientalis*/*buffeli*, and *B. major*) of cattle newly detected in 2013–2015 in Hungary [7]. This may support that the latter piroplasms might have been introduced recently into the country. On the other hand, seven further *Babesia*/*Theileria* species or genotypes were identified in this study that were hitherto unknown to occur in the region.

## Conclusions

This is the first simultaneous molecular investigation of piroplasms in representatives of all three *Haemaphysalis* spp., which occur in Central Europe. This report provides the first molecular evidence of piroplasms of small ruminants and cervids in Hungary, of *B. motasi* in Central-Eastern Europe, and of any *B. crassa*-like strain in Europe. The finding of *B. crassa* and *B. motasi* is also new in the tick species *H. inermis* and *H. concinna*, respectively. These data encourage scientists to broaden the scope of this study to other European countries.
